# Is there an effective way to control pain perception after free gingival graft removal? A systematic review and meta-analysis

**DOI:** 10.1590/0103-6440202305503

**Published:** 2023-12-22

**Authors:** Flavio X Ameida, Khalila C Cotrim, Eduardo C Kalil, Karen Bechara, Renan Dalla, Emanuel S Rovai, Jamil A Shibli, Nidia C Castro dos Santos

**Affiliations:** 1 Department of Periodontology, Dental Research Division, Guarulhos University, Guarulhos, SP, Brazil.; 2 Department of Dentistry, Dental Research Division, University of Taubaté, Taubaté, São Paulo, Brazil.; 3 Department of Periodontology, Institute of Science and Technology, São Paulo State University(UNESP), São José dos Campos, São Paulo, Brazil.; 4 The Forsyth Institute, The Forsyth InstituteCambridge, MA, United States

**Keywords:** Free Gingival Graft, Palatal Healing, Pain, Pain Control, Wound Healing

## Abstract

The literature describes multiple ways to stimulate wound healing to reduce the patient's perception of pain. This systematic review aimed to evaluate if methods that enhance wound healing can reduce the patient’s perception of pain after free gingival graft removal from the palate region compared to natural healing. A systematic review protocol was written following the PRISMA checklist. Electronic searches of five databases were performed to identify randomized clinical trials (RCTs) that assessed the patient’s perception of pain after the removal of a free gingival graft from the palate. The primary outcome was the visual analog scale (VAS) score assessing the patient’s perception of pain 7 days after the free gingival graft removal from the palate region. Of the 1,622 potentially relevant articles retrieved from the electronic databases, 16 RCTs were selected for qualitative analysis, and of these, 6 RCTs were included in the meta-analysis. RCTs showed a significant VAS reduction associated with the use of methods to enhance wound healing. The pooled estimates revealed a significant overall VAS reduction of 2.20 (95% CI 2.32, 2.07) 7 days after surgery. The methods that presented the greatest reduction in the perception of pain were platelet-rich fibrin, hyaluronic acid, and autologous fibrin glue. Methods that enhance wound healing, including platelet-rich fibrin, hyaluronic acid, and autologous fibrin glue, can reduce pain perception after free gingival graft removal in the palate region. However, only 1 RCT investigated each approach, which hinders the conclusion regarding the best procedure to reduce the perception of pain.

## Resumo

A literatura descreve diferentes formas de estimular a cicatrização para reduzir a percepção de dor do paciente. Esta revisão sistemática teve como objetivo avaliar se métodos que melhoram o reparo de feridas podem reduzir a percepção de dor do paciente após a remoção de enxerto gengival livre da região do palato quando comparado a cicatrização natural. Um protocolo de revisão sistemática foi escrito seguindo a lista de verificação PRISMA. Pesquisas eletrônicas em cinco bancos de dados foram realizadas para identificar ensaios clínicos aleatorizados (ECA) que avaliaram a percepção de dor do paciente após a remoção do enxerto gengival livre do palato. O desfecho primário foi o escore da escala visual analógica (VAS) avaliando a percepção de dor do paciente 7 dias após a remoção do enxerto gengival livre da região do palato. Dos 1.622 artigos potencialmente relevantes recuperados das bases de dados eletrônicas, 16 ECAs foram selecionados para análise qualitativa, e destes, seis ECAs foram incluídos na meta-análise. Os estudos analisados demonstraram uma redução significativa de VAS associada ao uso de métodos para melhorar a cicatrização de feridas. As estimativas agrupadas revelaram uma redução global significativa do VAS de 2,20 (95% CI 2,32, 2,07) 7 dias após a cirurgia. Os métodos que apresentaram maior redução na percepção de dor foram fibrina rica em plaquetas, ácido hialurônico e cola de fibrina autóloga. Métodos que melhoram a cicatrização de feridas podem reduzir a percepção de dor após a remoção do enxerto gengival livre na região do palato, especialmente fibrina rica em plaquetas, ácido hialurônico e cola de fibrina autóloga. No entanto, apenas um ECA avaliou cada abordagem, o que impossibilita a conclusão sobre qual é o melhor procedimento para reduzir a percepção de dor.

## Introduction

Soft tissue grafts have been widely used in clinical practice to increase tissue thickness (phenotype conversion), restore an adequate width of keratinized tissue, correct mucogingival deformities, and improve esthetics in teeth, and, more recently, in implants ^1^ ^,^ ^2^. A soft tissue graft harvested from the palate with the overlying epithelium is defined as a free gingival graft (FGG) and was first introduced to augment the keratinized tissue which was decreased or lost in one or more teeth ^3^. The importance of thick, keratinized soft tissue appears to be crucial for both natural teeth and dental implants ^4^. Just as teeth without keratinized attached gingiva, are more likely to present a greater loss of attachment ^5^, the deficiency of (or minimal) keratinized mucosa around the implants has been shown to hinder the patient's oral hygiene, leading to greater inflammation ^6^, mucosal recession and earlier bone loss^7^.

Likewise, a thick phenotype of soft tissue around teeth and implants is crucial in maintaining health and esthetics, with the use of connective tissue graft (CTG) contributing significantly to these outcomes ^1^ ^,^ ^4^ It has been speculated that approaches in which a deeper graft is removed from the palate could decrease its quality because it is richer in fatty and glandular tissue. On the other hand, the graft obtained by de-epithelization of an FGG would be composed of the lamina propria of the palate, a denser connective tissue with a greater amount of collagen fibers and, therefore, a better quality^8^. This would explain a greater increase in the thickness and quality of the buccal tissues after root coverage, in addition to being a more stable and easier-to-handle material, which would justify the use of this technique given the collection of deeper sub epithelial tissue graft.

The disadvantages of this procedure would be linked to the postoperative effects, with pain being the most common postoperative complication after palatal harvesting ^19^. Several approaches that claim to minimize patient morbidity, improve healing of palatal wounds, and reduce the perception of postoperative pain after FGG collection have been proposed, such as hyaluronic acid, platelet-rich fibrin (PRF) membranes, collagen sponge, use of cyanoacrylate, laser therapy ^10^ ^,^ ^11^ ^,^ ^12^ ^,^ ^13^

In this context, this systematic review aimed to evaluate if methods that enhance wound healing can reduce the patient’s perception of pain after free gingival graft removal from the palate region compared to natural healing. Thus, we aimed to answer the following PICOS question: “Does the use of methods that aim to enhance wound healing reduce the patient’s perception of pain after free gingival graft removal from the palate region compared to natural healing?”.

## Materials and methods

A systematic review protocol was written following the PRISMA checklist and was followed in both planning and reporting the review. The protocol was registered on 11/13/2022 with PROSPERO (available under ID: CRD42022360096).

This systematic review aimed to assess methods that enhance wound healing to reduce the patient’s perception of pain after free gingival graft removal from the palate region and compare it to natural healing.

The criteria used in this systematic review (SR) for the selection of studies were based on the PICOS method as follows:

- (P) Population: patients undergoing surgery to remove a free gingival graft from the palate region.
- (I) Interventions: methods that aim to enhance wound healing after free gingival graft removal from the palate region.
- (C) Comparison between interventions: natural healing.
- (O) Primary outcomes: assessment of pain after removal of free gingival graft in the palate region.
- (S) Type of studies: randomized controlled clinical trials.

Randomized clinical trials that included patients who were submitted to the removal of free gingival graft in the palate region were included in this review.

Studies that included patients with diabetes and studies that did not assess the patient’s pain perception. Studies that assessed sub epithelial connective grafts and studies that used alternative methods to control pain (*e.g.,* ozone therapy) were not included.

Methods aimed to improve wound healing and reduce pain perception after free gingival graft removal in the palate region (test groups) compared to natural healing (control group).

A visual analog scale (VAS) assessing pain perception was the primary outcome of this study. The secondary outcome was the consumption of analgesics.

Search strategies were developed at Pubmed/MEDLINE, Scopus, Cochrane Central Register of Controlled Trials, LILACS, and EMBASE databases up to November 2022. MeSH terms and keywords were combined to search the databases. The search strategy was as follows: ((palatal healing OR wound healing OR palatal wound OR palatal bandage OR palatal dressing) AND (free gingival graft OR palatal harvesting OR soft tissue graft)).

Two independent reviewers conducted the selection of the studies in the following steps:

1. . Initial screening of potentially suitable titles against inclusion criteria to identify potentially relevant articles (KB and KC). Before the initial screening, all items found through electronic searches were grouped into a single list, excluding duplicates using Rayyan software. ai (https://rayyan.ai/reviews/407327).
2. . Subsequently, two reviewers (EK and RD) independently examined the summaries (where available) of all reports identified in the single Rayyan list. The full article was obtained when studies met the inclusion criteria or when abstract data were insufficient to assess inclusion criteria.
3. . Eligibility of full articles identified as possibly relevant in the initial screening was made. Four reviewers (KB, KC, EK, and RD) independently assessed the full text of all studies of possible relevance.
4. . When any disagreement among the four reviewers was revealed, a consensus was reached by discussion between all reviewers.

All studies that met the inclusion criteria underwent quality assessment and data recording. A specifically designed standardized data extraction form was used to record data from each included study, covering article title, date, authors, number of patients, type of pain assessment; the technique used to remove the free gingival graft from the palate, data demographics, clinical methods (evaluation and treatment), time points of follow-up, and reported outcomes. At this stage, a decision was made between the reviewers (KB, KC, EK, and RD) for complete reading and data extraction performed independently. When any disagreement was detected between the reviewers, a consensus was reached by discussion between them.

The risk of bias in the included studies was evaluated according to the Cochrane Collaboration’s Tools for assessing the risk of bias. The quality assessment of performed independently by two reviewers (KC and KB), with disagreements resolved by a third adjudicator (NCCS). The randomization and selection methods (selection bias), completeness of the follow-up period/incomplete outcome data (attrition bias); blinding of patients (performance bias) and examiners (detection bias); selective reporting (reporting bias); and other forms of bias were classified as adequate (+), inadequate (−), or unclear (?). Based on these domains, the overall risk of bias was categorized as follows: ^1^ low risk of bias if all criteria were met (adequate methods of randomization and allocation concealment, a “yes” answer to questions about completeness of follow-up and blinding, and a “no” answer to selective reporting and other sources of bias); ^2^ unclear risk of bias if one or more criteria were partly met; or ^3^ high risk of bias if one or more criteria were not met.

The quality of evidence was verified based on the risk of bias, inconsistency, indirectness, and imprecision. The grades of Recommendation Assessment, Development, and Evaluation (GRADE) guidelines tool was used to assess the strength of evidence across clinical trials regarding the perception of pain.

Analyses were performed using the software Revman 5.4. Random-effects meta-analyses were conducted for VAS at 2, 3, 7, and 14 days post-treatment. Pooled outcomes were expressed as weighted mean differences (WMD). Statistical heterogeneity among the studies was assessed with the Cochrane Q statistic and *I* ^2^.

## Results

**Figure 1:**
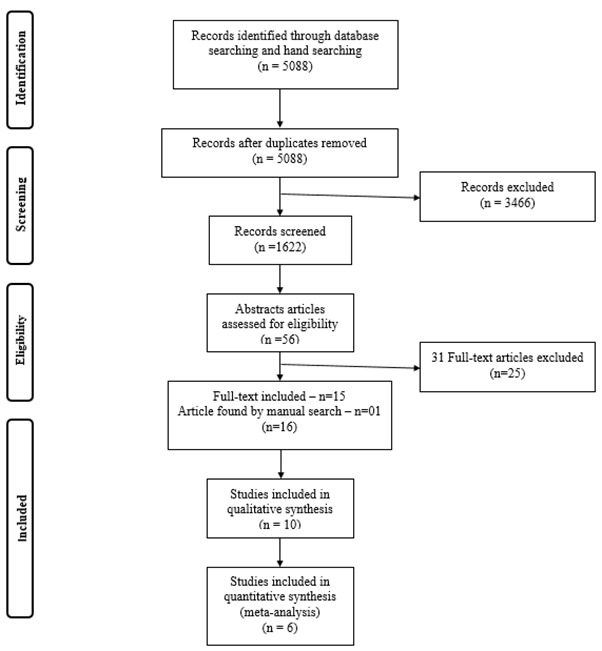
Flowchart

A total of 5,088 articles published between 2002 and 2022 were identified, evaluated, and selected according to inclusion criteria. Through the Rayyan software, 3,466 duplicate articles were detected and excluded. In addition, 1,622 articles were selected for reading titles, making the exclusion according to the selected criteria. Of this total, 56 were read in full, and 15 papers were selected for inclusion in this systematic review. One more article was included after a manual search in the literature. They were generating a total of 16 articles for reading and data extraction. The search in the gray literature did not generate new studies. The flow diagram of the search and selection process and the reasons for excluding potential studies are shown in Figure 1.

16 RCTs published between 2002 and 2022 met the eligibility criteria and were included in this literature review. Therefore, all 16 articles were included in the systematic review.

Table S1 shows the characteristics of the 16 studies included in the qualitative analysis. Overall, 613 participants were enrolled. The total follow-up varied between 3 days to 12 weeks. Regarding interventions, we observed more frequently the use of PRF, which includes 6 RCTs ^11^ ^,^ ^14^ ^,^ ^15^ ^,^ ^16^ ^,^ ^17^, and low-level laser therapy, ^12^ ^,^ ^13^ ^,^ ^19^. In eleven studies, the number of participants remained unchanged until the final follow-up ^10^ ^,^ ^11^ ^,^ ^12^ ^,^ ^13^ ^,^ ^14^ ^,^ ^15^ ^,^ ^16^ ^,^ ^17^ ^,^ ^18^ ^,^ ^19^ ^,^ ^20^ ^,^ ^21^ ^,^ ^22^. In the other 5 RCTs, 1 or more patients did not reach the final follow-up ^12^ ^,^ ^14^ ^,^ ^23^ ^,^ ^24^ ^,^ ^25^. All the studies included systemically healthy patients; 1 study included smokers ^21^.

In four studies, there was a difference between the groups regarding the use of analgesics, with a greater number of pills in the control group^11^ ^,^ ^18^ ^,^ ^19^ ^,^ ^23^ In four other studies, this difference was not found ^12^ ^,^ ^13^ ^,^ ^21^ ^,^ ^22^. In addition, eight other studies did not report the use of medication. ^10^ ^,^ ^14^ ^,^ ^15^ ^,^ ^16^ ^,^ ^17^ ^,^ ^20^ ^,^ ^24^ ^,^ ^25^

**Figure 2:**
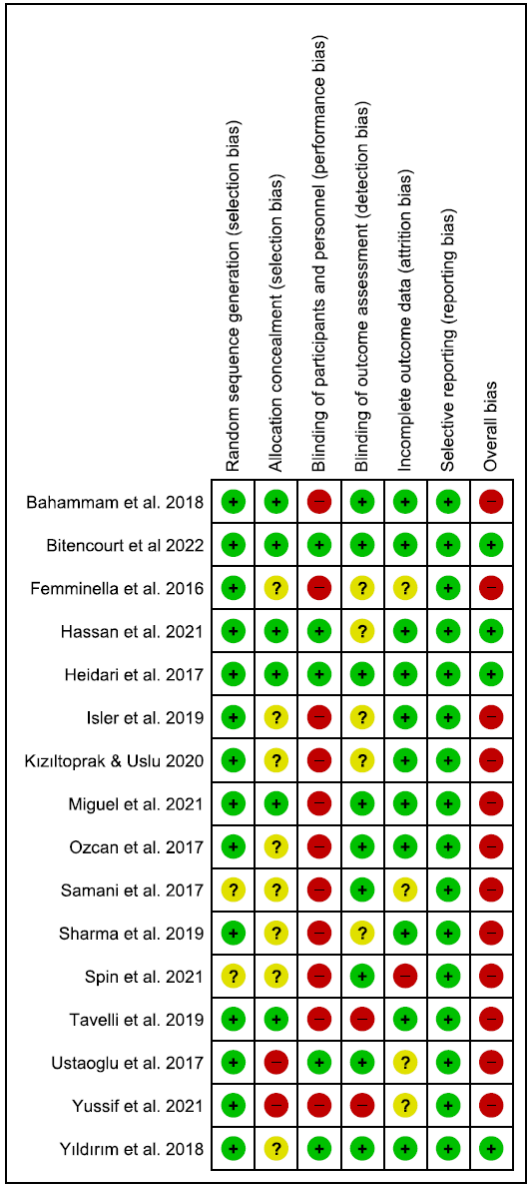
Risk of bias

Figure 2 presents the results of the risk of bias analysis. Two studies did not clarify the random sequence generation ^20^ ^,^ ^24^, and 7 studies did not adequately report the blinding of outcome assessment. ^11^ ^,^ ^16^ ^,^ ^17^ ^,^ ^18^ ^,^ ^21^ ^,^ ^23^ ^,^ ^25^

Overall, 4 RCTs had a low risk of bias ^13^ ^,^ ^19^ ^,^ ^23^ ^,^ ^25^, while 12 were considered to have a high risk of bias ^11^ ^,^ ^12^ ^,^ ^14^ ^,^ ^15^ ^,^ ^16^ ^,^ ^17^ ^,^ ^18^ ^,^ ^2021^ ^,^ ^22^ ^,^ ^24^ ^,^ ^25^.

Table 1 presents the GRADE summary for the quality of evidence. Six RCTs were included in the analysis ^10^ ^,^ ^11^ ^,^ ^17^ ^,^ ^18^ ^,^ ^20^ ^,^ ^22^. Risk of bias was considered “serious,” whilst Inconsistency, Indirectness, Imprecision, and Publication bias were considered “not serious.” Thus, the Quality of evidence was considered Moderate due to the high risk of bias.

**Figure 3:**
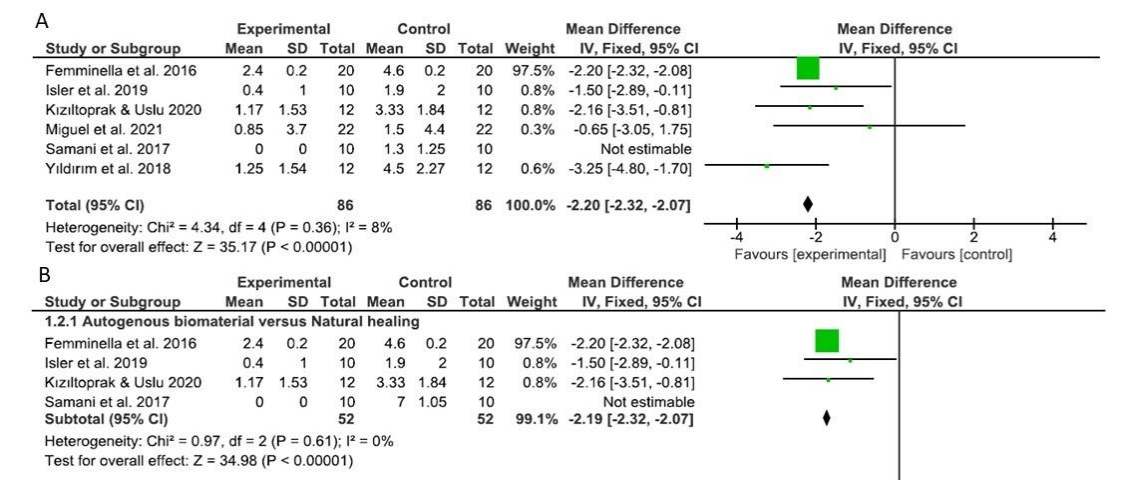
Meta-analysis

Meta-analyses were performed with data from 6 RCTs ^10^ ^,^ ^11^ ^,^ ^17^ ^,^ ^18^ ^,^ ^20^. Pooled estimates indicated significant differences between groups for VAS reduction (WMD: 2.20; 95% CI: 2.32,2.07; p < 0,00001; *i* ^*2*^ 8%) 7 days after surgery (Figure 3A). Subgroup analysis for the origin of biomaterial showed a significant effect of treatment for VAS reduction for autogenous (WMD: 2.19; 95% CI: 2.32,2.07; p < 0,00001; *i* ^*2*^ 0%) and xenogenous (WMD: 2.48; 95% CI: 3.79,1.18; p = 0,0002; *i* ^*2*^ 69%) biomaterials 7 days after surgery (Figure 3B). When different follow-ups were analyzed, experimental treatment reduced mean VAS at 2 (WMD: 2.82; 95% CI: 3.59,2.04; p < 0,00001; *i* ^*2*^ 85%), 3 (WMD: 3.04; 95% CI: 3.92,2.16; p < 0,00001; *i* ^*2*^ 83%), and 14 (WMD: 1.99; 95% CI: 2.13,1.86; p < 0,00001; *i* ^*2*^ 0%) days post-surgery.

**Table 1:** GRADE summary of findings table for the methods to enhance wound healing compared with natural healing 7 days after free gingival graft removal from the palate region

Certainty assessment							Effect	Quality of Evidence	Key message
№ of studies	Study design	Risk of bias	Inconsistency	Indirectness	Imprecision	Publication bias	Mean Difference (95% CI)		
6	Randomized trial	Serious ^a^	Not serious ^b^	Not serious ^c^	Not serious ^d^	Not serious ^e^	-2.2 VAS (95% CI -2.32, -2.07)	MODERATE Due to high risk of bias.	Methods that enhance wound healing probably reduce the perception of pain after free gingival graft removal from the palate region.

## Discussion

This systematic review identified 16 clinical trials that assessed the use of methods to enhance wound healing after free gingival graft removal from the palate region. Of these, 6 studies were included in the meta-analysis. The results of the meta-analysis showed that using methods that enhance wound healing significantly reduced VAS scores compared with natural healing 7 days after free gingival graft removal. This was the first systematic review and meta-analysis that assessed methods that aim to reduce the patient’s perception of pain after free gingival graft removal from the palate region. In a subgroup analysis considering different types of biomaterials, VAS was also decreased with the use of either autogenous or xenogenous biomaterials 7 days after surgery.

We carried out additional analysis according to the biomaterials tested in the selected studies. 4 RCTs used autogenous biomaterials to enhance wound healing in the palate donor region, ^11^ ^,^ ^17^ ^,^ ^18^ ^,^ ^20^. These included platelet-rich derivate, such as PRP, PRF, and AFG. As for the xenogenous biomaterials, 1 RCT used EMD ^22^ and 1 RCT used HA ^10^ in the palate region after free gingival graft removal. In both scenarios, VAS scores decreased after 7 days. Autogenous and xenogenous biomaterials promoted similar outcomes in the reduction of pain perception. 

Another topic of interest regarding pain perception is the moment the patient perceives it. Thus, we conducted additional analyses for different follow-ups assessed in the clinical trials. As a result, we observed that other treatments to enhance wound healing in the donor site promote the most significant reductions of pain as perceived by the patients at 2 and 3 days of follow-up. Even though this finding is expected, as the acute inflammatory process tends to exacerbate the perception of pain in the first 72h (Inflammatory phase), our results encourage the clinician to choose an additional treatment for the donor area instead of expecting spontaneous healing alone, since methods to enhance wound healing can help to reduce discomfort when the patient is more vulnerable. Fourteen days after free gingival graft removal, pain perception was greater when a therapy to improve wound healing was applied. Interestingly, one study reported that the mean VAS score was zero for the experimental and the control groups, suggesting that, eventually, both approaches might reach similar standards.

The outcome assessed in this meta-analysis was a patient-centered outcome measure (PROM). Regulatory agencies, including the Food and Drug Administration (FDA), have suggested that clinically meaningful outcomes should be able to measure how a patient feels (e.g. symptoms) directly, functions, or survives, and such endpoints should be reported in clinical trials. Even though PROMs can be subjective results of clinical procedures, assessing pain using VAS and other PROMs should be considered in future studies that evaluate methods to improve wound healing since the most clinically meaningful goals of such procedures are to reduce pain symptoms and increase the quality of life.

Nevertheless, as for any other PROM, pain perception can be subjective. Thus, the use of the VAS scale needs to be standardized in future studies. In addition, the prescription of analgesics following ethical guidelines should be reported.

As a secondary variable, we assessed the use of analgesics in the control and test groups of the included studies. There was great heterogeneity in the medication regimen among the studies, which precluded a meta-analysis. Among the RCTs included in the meta-analysis, only 2 (11, 18) reported greater use of analgesics by the participants in the control group. Thus, even with the use of medication to control pain, the test groups presented a reduction in VAS scores, revealing that methods to enhance wound healing surpassed the use of analgesics in the control of pain. For the other RCTs, there was no difference between the groups, or the authors did not report the use of analgesics at all.

In this systematic review, the risk of bias was assessed according to the Cochrane Collaboration tool. Most studies were considered to have a high chance of bias ^11^ ^,^ ^12^ ^,^ ^14^ ^,^ ^15^ ^,^ ^16^ ^,^ ^17^ ^,^ ^18^ ^,^ ^20^ ^,^ ^22^ ^,^ ^24^ ^,^ ^25^. The domain that mainly impacted these studies was the blinding of the participants. Nonetheless, the high risk of bias found in most of the trials also affected the GRADE assessment, downgrading the quality of evidence to Moderate.

For procedures that require blood collection, the high risk of bias is inherent to the trial methodology, since collecting blood from the patients in the control group would not be ethically acceptable. However, future studies that use xenogenous biomaterials could reduce their risk of bias by using a placebo in the control group.

Large varieties of procedures that reduce pain perception after free gingival graft removal from the palate have been reported. The methods that presented the greatest reduction in the perception of pain were platelet-rich fibrin, hyaluronic acid, and autologous fibrin glue. Methods that enhance wound healing, including platelet-rich fibrin, hyaluronic acid, and autologous fibrin glue, can reduce pain perception after free gingival graft removal in the palate region. However, only 1 RCT investigated each approach, which hinders the conclusion regarding the best procedure to reduce the perception of pain.

## Figures and Tables

**Supplementary Table1:**
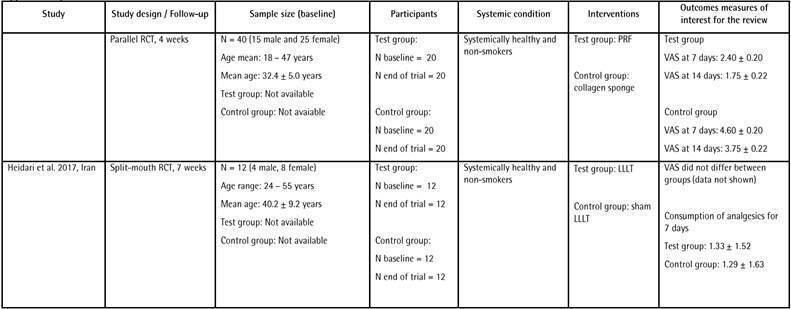
. Characteristics of the included studies

**SupplementaryTable 1:**
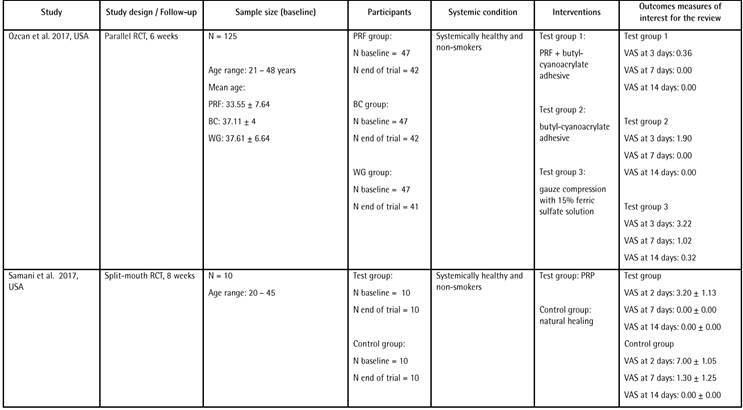
Continuation

**Supplementary Table 1:**
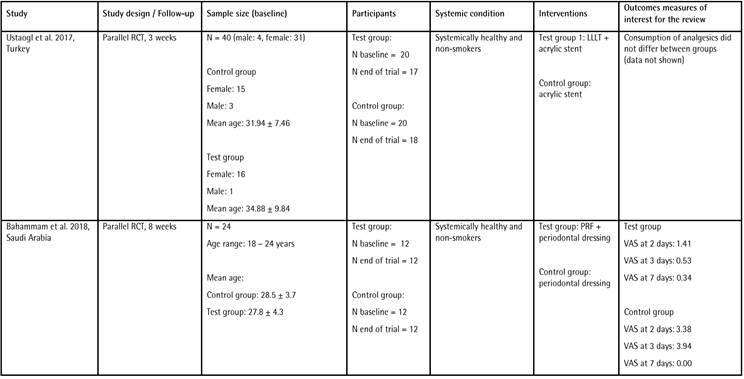
Continuation

**SupplementaryTable 1:**
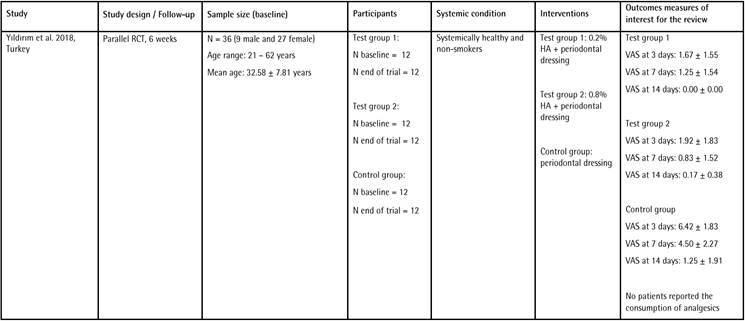
Continuation

**SupplementaryTable 1.**
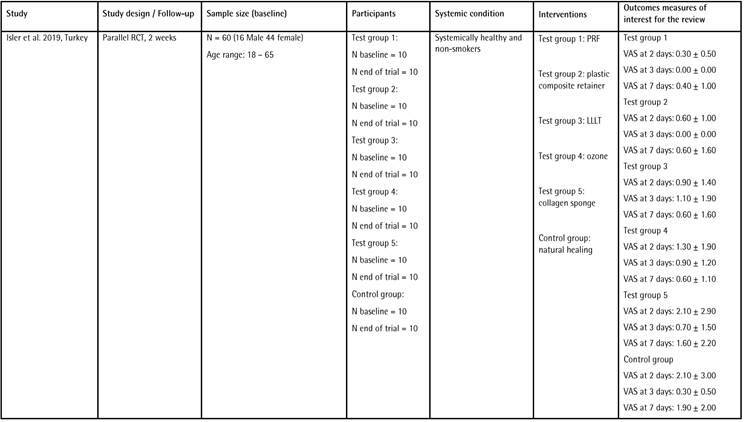
Continuation

**SupplementaryTable 1:**
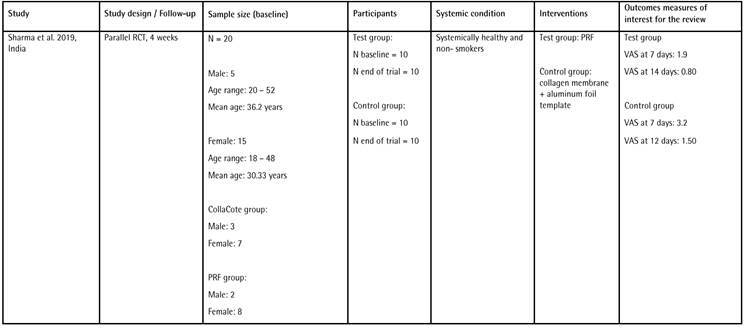
Continuation

**SupplementaryTable 1:**
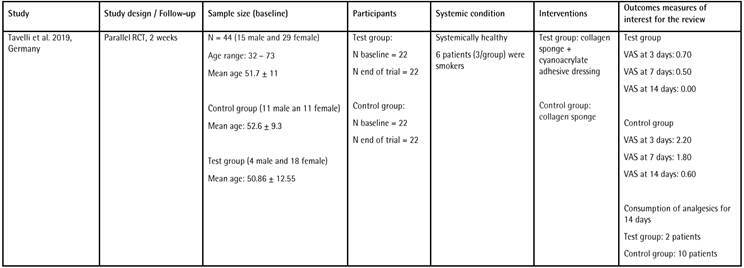
Continuation

**SupplementaryTable 1:**
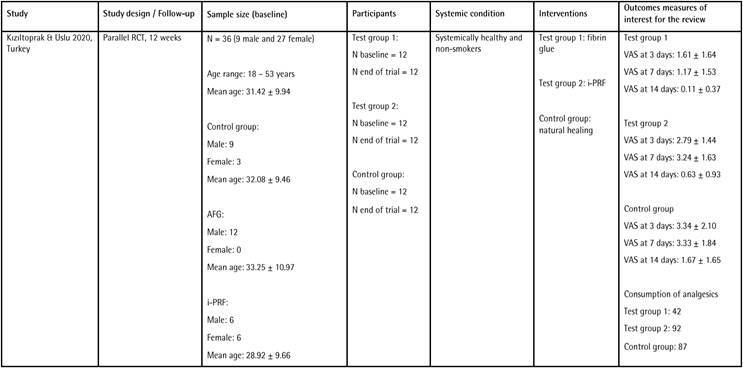
Continuation

**SupplementaryTable 1:**
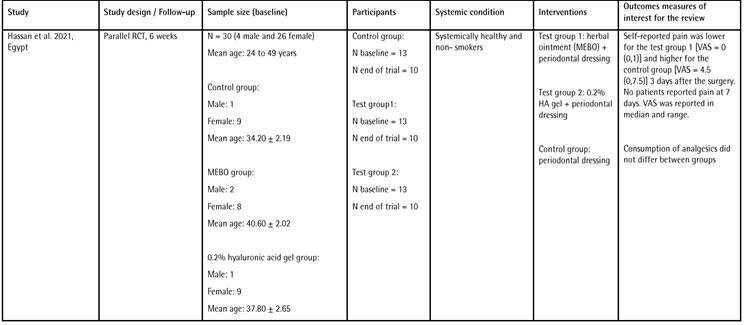
Continuation

**Supplementary Table 1:**
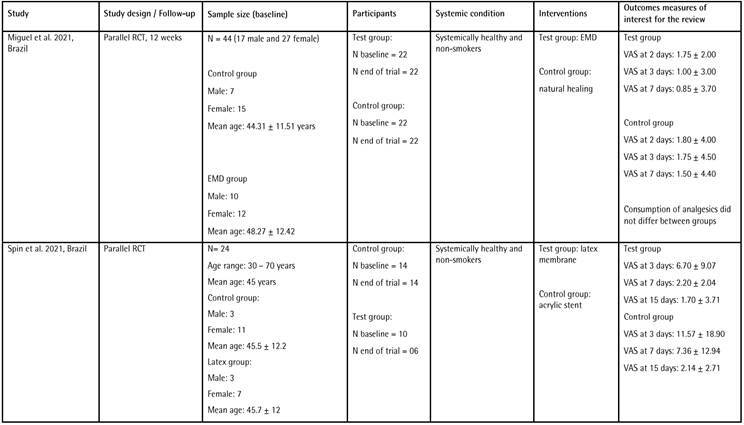
Continuation

**SupplementaryTable 1.**
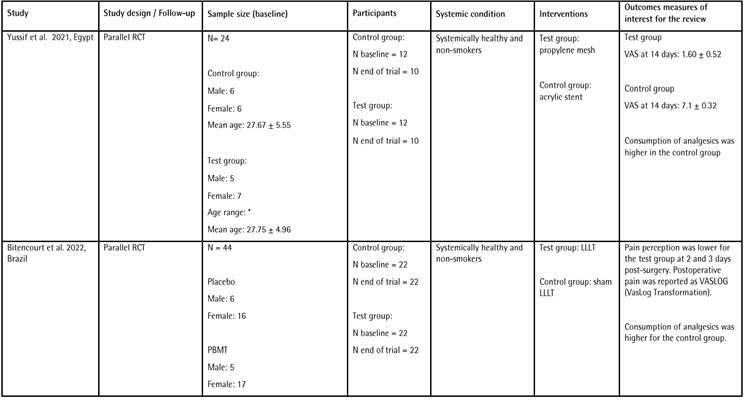
Continuation

## References

[1] Zucchelli G, Mounssif I (2015). Periodontal plastic surgery. Periodontol.

[2] Dias SBF, Fonseca MVA, dos Santos NCC (2015). Effect of GaAIAs low-level laser therapy on the healing of human palate mucosa after connective tissue graft harvesting: a randomized clinical trial. Lasers Med Sci.

[3] Nabers JM (1966). Free gingival grafts. Periodontics.

[4] Zucchelli G, Felice P, Mazzotti C (2018). 5-year outcomes after coverage of soft tissue dehiscence around single implants: A prospective cohort study. Eur J Oral Implantol.

[5] Chambrone L, Tatakis DN (2016). Long-Term Outcomes of Untreated Buccal Gingival Recessions: A Systematic Review and Meta-Analysis. J Periodontol.

[6] Lin G-H, Chan H-L, Wang H-L (2013). The significance of keratinized mucosa on implant health: a systematic review. J Periodontol.

[7] Bengazi F, Botticelli D, Favero V (2014). Influence of presence or absence of keratinized mucosa on the alveolar bony crest level as it relates to different buccal marginal bone thicknesses. Clin Oral Implants Res.

[8] Bertl K, Pifl M, Hirtler L (2015). Relative Composition of Fibrous Connective and Fatty/Glandular Tissue in Connective Tissue Grafts Depends on the Harvesting Technique but not the Donor Site of the Hard Palate. J Periodontol.

[9] Tavelli L, Barootchi S, Di Gianfilippo R (2021). Patient experience of autogenous soft tissue grafting has an implication for future treatment: A 10- to 15-year cross-sectional study. J Periodontol.

[10] Yıldırım S, Özener HÖ, Doğan B, Kuru B (2018). Effect of topically applied hyaluronic acid on pain and palatal epithelial wound healing: An examiner-masked, randomized, controlled clinical trial. J Periodontol.

[11] Femminella B, Iaconi MC, Di Tullio M (2016). Clinical Comparison of Platelet-Rich Fibrin and a Gelatin Sponge in the Management of Palatal Wounds After Epithelialized Free Gingival Graft Harvest: A Randomized Clinical Trial. J Periodontol.

[12] Ustaoglu G, Ercan E, Tunali M (2017). Low-Level Laser Therapy in Enhancing Wound Healing and Preserving Tissue Thickness at Free Gingival Graft Donor Sites: A Randomized, Controlled Clinical Study. Photomed Laser Surg.

[13] Heidari M, Paknejad M, Jamali R (2017). Effect of laser photobiomodulation on wound healing and postoperative pain following free gingival graft: A split-mouth triple-blind randomized controlled clinical trial. J Photochem Photobiol B.

[14] Ozcan M, Ucak O, Alkaya B (2017). Effects of Platelet-Rich Fibrin on Palatal Wound Healing After Free Gingival Graft Harvesting: A Comparative Randomized Controlled Clinical Trial. Int J Periodontics Restorative Dent.

[15] Bahammam MA (2018). Effect of platelet-rich fibrin palatal bandage on pain scores and wound healing after free gingival graft: a randomized controlled clinical trial. Clin Oral Investig.

[16] Sharma V, Kumar A, Puri K (2019). Application of platelet-rich fibrin membrane and collagen dressing as palatal bandage for wound healing: A randomized clinical control trial. Indian J Dent Res.

[17] Işler SÇ, Uraz A, Şengül J (2019). Evaluation of the patient's oral health related quality of life after harvesting free gingival graft. Cumhuriyet Dental Journal.

[18] Kızıltoprak M, Uslu MÖ (2020). Comparison of the effects of injectable platelet-rich fibrin and autologous fibrin glue applications on palatal wound healing: a randomized controlled clinical trial. Clin Oral Investig.

[19] Bitencourt FV, Cardoso De David S, Schutz J da S (2022). Minimizing patient morbidity after free gingival graft harvesting: A triple-blind randomized-controlled clinical trial. Clin Oral Implants Res.

[20] Samani MK, Saberi BV, Ali Tabatabaei SM, Moghadam MG (2017). The clinical evaluation of platelet-rich plasma on free gingival graft’s donor site wound healing. Eur J Dent.

[21] Tavelli L, Ravidà A, Saleh MHA (2019). Pain perception following epithelialized gingival graft harvesting: a randomized clinical trial. Clin Oral Investig.

[22] Miguel MMV, Mathias-Santamaria IF, Rossato A (2021). Enamel matrix derivative effects on palatal mucosa wound healing: Randomized clinical trial. J Periodontal Res.

[23] Hassan A, Ahmed E, Ghalwash D, Elarab AE (2021). Clinical Comparison of MEBO and Hyaluronic Acid Gel in the Management of Pain after Free Gingival Graft Harvesting: A Randomized Clinical Trial. Int J Dent.

[24] Spin JR, de Oliveira GJPL, Spin-Neto R (2021). Effect of natural latex membranes on wound repair of palate donor areas: A pilot randomized controlled trial study, including the membranes characterization. Mater Today Commun.

[25] Yussif N, Wagih R, Selim K (2021). Propylene mesh versus acrylic resin stent for palatal wound protection following free gingival graft harvesting: a short-term pilot randomized clinical trial. BMC Oral Health.

